# The Role of Tryptophan-Kynurenine in Feather Pecking in Domestic Chicken Lines

**DOI:** 10.3389/fvets.2019.00209

**Published:** 2019-06-27

**Authors:** Patrick Birkl, Jacqueline Chow, Paul Forsythe, Johanna M. Gostner, Joergen B. Kjaer, Wolfgang A. Kunze, Peter McBride, Dietmar Fuchs, Alexandra Harlander-Matauschek

**Affiliations:** ^1^Department of Animal Biosciences, University of Guelph, Guelph, ON, Canada; ^2^Department of Medicine, Brain-Body Institute and Firestone Institute for Respiratory Health, McMaster University, Hamilton, ON, Canada; ^3^Division of Medical Biochemistry, Medical University Innsbruck, Biocenter, Innsbruck, Austria; ^4^Friedrich-Loeffler-Institut, Federal Research Institute for Animal Health, Institute of Animal Welfare and Animal Husbandry, Celle, Germany; ^5^Division of Biological Chemistry, Medical University Innsbruck, Biocenter, Innsbruck, Austria

**Keywords:** tryptophan, immune system, IDO, neopterin, problem behavior

## Abstract

Research into the role of tryptophan (TRP) breakdown away from the serotonergic to the kynurenine (KYN) pathway by stimulating the brain-endocrine-immune axis system interaction has brought new insight into potential etiologies of certain human behavioral and mental disorders. TRP is involved in inappropriate social interactions, such as feather-destructive pecking behavior (FP) in birds selected for egg laying. Therefore, our goal was to determine the effect of social disruption stress on FP and the metabolism of the amino acids TRP, phenylalanine (PHE), tyrosine (TYR), their relevant ratios, and on large neutral amino acids which are competitors with regard to their transport across the blood-brain barriers, at least in the human system, in adolescent birds selected for and against FP behavior. We used 160 laying hens selected for high (HFP) or low (LFP) FP activity and an unselected control line (UC). Ten pens with 16 individuals each (4 HFP birds; 3 LFP birds; 9 UC birds) were used. At 16 weeks of age, we disrupted the groups twice in 5 pens by mixing individuals with unfamiliar birds to induce social stress. Blood plasma was collected before and after social disruption treatments, to measure amino acid concentrations. Birds FP behavior was recorded before and after social disruption treatments. HFP birds performed significantly more FP and had lower KYN/TRP ratios. We detected significantly higher FP activity and significantly lower plasma PHE/TYR ratios and a trend to lower KYN/TRP ratios in socially disrupted compared to control pens. This might indicate that activating insults for TRP catabolism along the KYN axis in laying hens differs compared to humans and points toward the need for a more detailed analysis of regulatory mechanisms to understand the role of TRP metabolism for laying hen immune system and brain function.

## Introduction

Birds selected for egg-laying face many serious animal welfare problems. One of the most challenging issues is feather pecking (FP). Feather pecking (FP) is a repetitive oral behavior in which individuals peck repetitively at another bird's feather cover (1). The act often results in feathers or parts of feathers being broken off, plucked/pulled (2) and subsequently ingested (3–5), causing significant skin injuries to the victim (6). Hence, FP in birds selected for egg laying takes place in a social context between two birds.

On commercial farms, these birds live in a densely populated environment with large groups of thousands of female individuals (7). Under natural conditions, social groups typically consist of one male with up to 20 females (8, 9) demonstrating the dramatic contrast in group size. In these groups, it is unlikely for birds to adequately recognize/remember each other. These social environments constantly change. More specifically, a social environment such as this may have strong adverse effects on behavioral functioning, producing abnormally high levels of inappropriate social behavior such as FP (10, 11). In particular, FP behavior may naturally shape the social environment of the involved birds, where feather peckers may cause social stress and social stress causes FP within a group due to social instability (12, 13). FP may also be influenced by the social rank of an individual within a social group (14). Moreover, feather peckers appear less social by displaying less motivation to join a group (15) and have pronounced stress reactions to increased social contact (16).

The neurobiological mechanisms that underlie FP are not well-understood. It has been suggested that the avian serotonergic (5-HT) system (17) and the precursor tryptophan (TRP) are intimately linked to repetitive FP. Furthermore, genetic factors (18) such as candidate genes linked to the 5-HT system (19), lower forebrain 5-HT turnover levels (20, 21), and nutritional factors, including low dietary TRP (22) also point toward serotonergic system involvement in the development of FP behavior.

In the human, it is well-known that stressful situations can alter peripheral and brain TRP levels by stimulating the immune system and activating the HPA-axis (23). Therefore, the concept of TRP metabolism alterations elicited by stressful events is of growing interest for understanding TRP/5-HT-related behavioral disorders, characterized by dysfunction in social interactions in humans, such as depression (24) or autism-spectrum disorders (25). In the human, TRP is degraded in two major pathways: the kynurenine (KYN) pathway and the 5-HT/serotonergic pathway. Cytokines activate indolamine 2,3-dioxygenase-1 (IDO-1), converting TRP to KYN. The same conversion is achieved by enzyme IDO-2 with a different activity rate and activation pattern (26). Another TRP catabolic enzyme is tryptophan-2,3-dioxygenase (TDO), expressed mainly in liver, kidney, and brain, which is activated by the substrate itself or by hormones (glucocorticoids, prolactin) (27). The second pathway, the 5-HT/serotonergic pathway, is initiated by TRP hydroxylase (TPH) (28). Stressful events activating TDO and immunological challenges inducing IDO activity, which metabolizes TRP to KYN, may be depleting the system of TRP and attenuate to produce 5-HT in the mammalian brain (29). Additionally, since 5-HT cannot cross the blood-brain barrier, central 5-HT synthesis depends on the availability and transport of its precursor, TRP, into the brain (30). TRP competes with other large neutral amino acids (LNAAs) for active transport by the large amino acid transporter system (31). Consequently, decreased peripheral TRP relative to other LNAAs results in a lower TRP influx into the central nervous system. The KYN/TRP ratio reflects mainly IDO-1 activity in the human system when additional proinflammatory markers such as neopterin are present (32). Alterated KYN/TRP ratios were found in people with major depressive disorders and in patients with chronic disorders additionally suffering from depression and reduced quality of life (33, 34). Additional to TRP metabolism, in the human system, pro-inflammatory cascades were found to be associated with disturbed phenylalanine hydroxylase (PAH) activity (35), which metabolizes phenylalanine (PHE) to tyrosine (TYR) and is reflected by the PHE/TYR ratio. Tyrosine is further catabolized to the neurotransmitters adrenalin, noradrenalin, and dopamine (DA). In our previous work, we have established that PAH activity (measured by the ratio of PHE/TYR) may play a role in the development of injurious head pecking behavior, while FP was not observed (36).

The present study aimed to investigate the effect of social stress resulting from social disruption on (i) FP activity, (ii) metabolism of the amino acids TRP, PHE, TYR, their relevant ratios, and (iii) related biomarkers of immune activation in adolescent birds selected for and against FP behavior. We hypothesized that social disruption treatment is associated with increased FP, an accelerated TRP metabolism, shifts to PHE/TYR metabolism, and increased neopterin production. Neopterin was measured as it is a sensitive marker of immune activation in humans and primates, where its formation is mainly activated by interferon-γ alongside IDO-1 activity (37) and because of the relationship between psychological stressors and immune system activation (38).

## Materials and Methods

### Ethical Statement

This study was approved by the University of Guelph Animal Care Committee before testing (Animal Utilization Protocol # 3206).

### Animals and Housing

The birds in the present study originated from a selection experiment in which birds were divergently selected for based on FP activity, high (HFP) and low (LFP), and an unselected control line (UC) (18). In these pedigree lines, the mating of close relatives was avoided. Twenty male and 40 female breeders in the UC line and ten male and 25 female breeders in the selected lines were used to producing the chicks. One-day-old non-beak trimmed chicks were imported from Europe and arrived individually wing-tagged (with pedigree information). The experimenters were blinded to the lines. Because the numbers of female chicks per line were unequal, the genetic lines were mixed in with the groups of the experimental set-up to create an equal genetic environment in each experimental pen. Ten pens with 16 individuals each (4 HFP birds; 3 LFP birds; 9 UC birds) were used. The floor pens were identical to the one described in Kozak et al. (39). Visual barriers between the pens were provided. Pens offered space for nine adult birds/m^2^, 15 cm perch length/bird at 90 cm above the ground, 125 × 31 cm plastic slatted platforms 65 cm above the ground, 10 cm food trough length/bird, and ten nipple drinkers. Wood shavings were used as floor bedding material. Cameras (Samsung SNO-5080R, IR, Samsung Techwin CO., Gyeongi-do Korea) were ceiling-mounted within each pen above the entrance. The birds were kept in a ventilated, windowless room with a light intensity of 25 lx at animal level. Management of the temperature and lighting schedule during rearing was according to commercial management guidelines.

### Social Disruption Stress Treatment

Ten days before the social disruption treatment, the birds (14 weeks of age) were individually tagged with numbered soft silicone plates fastened to the backs of the birds using elastic straps around their wings, which served to identify birds on video observations (40). At 16 weeks of age, we disrupted five pens by mixing individuals with unfamiliar birds from other pens to induce social stress, while the other five pens remained undisrupted (control groups/pens). For each social disruption treatment, each pen was split into four sub-groups of four individuals per sub-group (1 HFP, 1 LFP, 2 UC). The sub-groups were taken out of their five “home” pens and distributed into four other pens where they encountered 12 unfamiliar individuals in each new pen. This resulted in the new pen consisting of 4 sub-groups of 4 individuals each, with each sub-group deriving from a different pen (*n* = 16 individuals per pen). Three days after social disruption treatment, the procedure was repeated to increase the effects of social stress by disrupting the newly established social environment. The genetic make-up of the groups of 16 remained identical throughout the entire experiment (4 HFP birds; 3 LFP birds; 9 UC birds). Control groups were caught and released into a different pen (group remained undisrupted) as a sham treatment. In summary, five treatment groups received social disruption treatment, and five controlled groups received no social disruption treatment. All groups were moved out of their home pens to avoid “home pen” advantage.

### Behavioral Observations

Behavioral observations of each pen were undertaken 2 days before social disruption treatment to record baseline (pre-treatment) behavior [10 min in the morning (9:00 h) and 10 min in the afternoon (14:00 h)]. We chose our observation length based on previous research with FP video-observations [e.g., (41)]. Post-treatment we recorded 2 minutes (for a period of 10 min), 1 h (for a period of 10 min) and on the following day [10 min in the morning (9:00 h) and 10 min in the afternoon (14:00 h)]. When two observations were conducted on the same day, we averaged FP occurrence across both observations. In sum, to evaluate FP levels, each individual bird was observed for 60 min: two 10 min baseline observations and four 10 min post-disruption observations. During the observation periods, all occurrences (42) of FP were recorded. FP was subdivided into gentle and severe FP pecks as described in Bilčik and Keeling (10).

### Body Weight

Each birds' bodyweight was measured 1 day before (at 16 weeks of age) and 3 weeks post (at 19 weeks of age) the social disruption stress.

### Blood Measurements

Blood samples were taken from each bird 1 day before the first treatment (baseline data) and 4 days after the second social disruption treatment. The wing vein of each bird was punctured with a 21-gauge butterfly needle to draw three mL of blood per animal into an EDTA tube. All blood collections were performed between 9:00 and 12:00 h. Individual birds were sampled at the same time point on both blood collection days.

Blood samples were stored on ice until centrifuged for 10 min at 4°C and 2,500 rpm for plasma separation. Plasma was then aliquoted into 1.5 mL vials and stored at −20°C until shipped for aromatic amino acid (AAA), Tryptophan (TRP), Tyrosine (TYR), Phenylalanine (PHE), and large neutral amino acids (LNAA); Threonine, Methionine, Valine, Leucine, Isoleucine, and Histidine analysis performed on a Waters Acquity Ultra Performance Liquid Chromatography (UPLC) system (Waters, Manchester, UK) at the SickKids Proteomics, Analytics, Robotics & Chemical Biology Centre at the SickKids Hospital, Toronto, Canada.

The remaining plasma samples were used to analyze the concentrations of free TRP and KYN as well as concentrations of PHE and TYR by high-performance liquid chromatography (HPLC) on a ProStar Varian system (USA) using rp-18 columns (LiChroCART 55-4, 3 μm grain size; Merck, Germany) and acetic-sodium acetate buffer (pH = 4) as eluent (flow-rate: 0.9 mL/min) according to the protocol described earlier (43) at the Biocentre of the Medical University in Innsbruck, Austria. Three-nitro-L-TYR (Sigma Aldrich, Austria) was used as an internal standard. TRP and KYN standards were purchased from Sigma-Aldrich (Austria). KYN and 3-nitro-L-TYR were detected by UV-absorbance at 360 nm wavelength (Shimadzu SPD-6A UV detector, Austria). TRP was detected by its fluorescence with an excitation wavelength of 286 nm and an emission wavelength of 366 nm (ProStar 360 detector, Varian, USA). The sensitivity of the measurements was 0.1 μmol/L TRP and 0.5 μmol/L KYN. HPLC and UPLC methods of amino acid measurements showed strong positive correlations [*r* = 0.61; see (44)]. Neopterin was measured by ELISA with a detection limit of 2.0 nmol/L (BRAHMS Diagnostics, Hennigdorf, Germany).

### Statistical Analysis

To analyze the effect of social disruption stress on behavior and physiology, we used a Proc Glimmix procedure in SAS (SAS Institute Inc., Cary, NC, 2016).

The effect of social disruption stress treatment (disruption/no disruption) and line (HFP, LFP, UC), and their interactions on FP behavior, body weight and blood measurements as the response variable, with averaged baseline measurements before the treatment as covariates, was measured. In the random statement, we accounted for repeated measurements on pens and mixing the lines within the pens. We used Poisson distribution for behavioral data, a Gaussian distribution for body weight and blood measurements, and a compound symmetry structure was fitted to the model. The degrees of freedom were adjusted using the Kenward-Roger method. Due to zero-inflated pecking data (counts), gentle FP and severe FP were merged into one category. Normal distribution of residuals was tested by employing a Proc Univariate procedure. Data are presented as LSM ± SE unless otherwise stated.

## Results

Descriptive statistics for biological markers before (PRE) and after (POST) social disruption treatment in disrupted and undisrupted groups across all lines and separately for each line are listed in Tables 1, 2.

**Table 1:** Descriptive statistics (mean ± standard deviation) for biological markers before (PRE) and after (POST) social disruption treatment in disrupted and undisrupted groups.

	**Undisrupted baseline PRE**	**Undisrupted POST**	**Disrupted baseline PRE**	**Disrupted POST**
Tryptophan (μmol/L)	105.69 ± 9.20	98.50 ± 13.13	101.13 ± 12.64	98.32 ± 10.65
Kynurenine (μmol/L)	0.34 ± 0.09	0.35 ± 0.08	0.30 ± 0.09	0.36 ± 0.29
Kyn/Trp (μmol/mmol)	32.48 ± 0.784	35.50 ± 0.76	28.65 ± 0.793	31.88 ± 0.89
Neopterin (nmol/L)	2.7 ± 0.42	2.74 ± 0.44	2.88 ± 0.48	2.81 ± 0.60
Tyrosine (μmol/L)	199.87 ± 44.40	197.75 ± 41.72	208.38 ± 36.45	207.12 ± 32.54
Phenylalanine (μmol/L)	133.08 ± 22.26	135.37 ± 21.05	133.73 ± 13.79	132.15 ± 11.85
Phe/Tyr (mol/mol)	0.67 ± 0.13	0.71 ± 0.11	0.63 ± 0.09	0.62 ± 0.09
TRP/AAA	0.36 ± 0.05	0.30 ± 0.04	0.33 ± 0.04	0.28 ± 0.03
TRP/LNAA	0.09 ± 0.01	0.09 ± 0.01	0.09 ± 0.01	0.09 ± 0.01

**Table 2:** Descriptive statistics (mean ± standard deviation) for biological markers before (PRE) and after (POST) social disruption treatment in high (HFP), low (LFP), and unselected control (UC) lines in disrupted and undisrupted groups.

**UC line**	**Undisrupted baseline PRE** **(*n* = 45)**	**Undisrupted POST** **(*n* = 45)**	**Disrupted baseline PRE** **(*n* = 45)**	**Disrupted POST** **(*n* = 45)**
Tryptophan (μmol/L)	104.03 ± 9.23	96.76 ± 13.93	103.64 ± 10.29	99.78 ± 10.14
Kynurenine (μmol/L)	0.34 ± 0.08	0.35 ± 0.08	0.30 ± 0.09	0.40 ± 0.39
Kyn/Trp (μmol/mmol)	33.0 ± 7.3	36.0 ± 8.22	29.3 ± 7.6	34.6 ± 10.3
Neopterin (nmol/L)	27.0 ± 4.2	31.8 ± 5.8	29.6 ± 6.8	32.2 ± 2.4
Tyrosine (μmol/L)	204.79 ± 29.35	204.73 ± 35.41	221.07 ± 32.60	220.36 ± 32.45
Phenylalanine (μmol/L)	134.10 ± 19.91	136.63 ± 18.83	140.36 ± 13.53	138.72 ± 12.14
Phe/Tyr (mol/mol)	0.66 ± 0.08	0.67 ± 0.07	0.64 ± 0.07	0.64 ± 0.08
TRP/AAA	0.34 ± 0.04	0.28 ± 0.03	0.33 ± 0.03	0.28 ± 0.03
TRP/LNAA	0.09 ± 0.01	0.09 ± 0.01	0.10 ± 0.01	0.09 ± 0.01
**LFP line**	**Undisrupted baseline PRE** **(*n* = 15)**	**Undisrupted POST** **(*n* = 15)**	**Disrupted baseline PRE** **(*n* = 15)**	**Disrupted POST** **(*n* = 15)**
Tryptophan (μmol/L)	110.79 ± 8.57	102.87 ± 11.08	105.60 ± 17.99	105.33 ± 12.07
Kynurenine (μmol/L)	0.34 ± 0.08	0.36 ± 0.09	0.28 ± 0.07	0.32 ± 0.09
Kyn/Trp (μmol/mmol)	30.6 ± 6.6	35.3 ± 7.4	26.3 ± 5.4	30.5 ± 7.1
Neopterin (nmol/L)	26.9 ± 3.4	31.3 ± 4.2	28.8 ± 0.50	31.1 ± 2.7
Tyrosine (μmol/L)	220.87 ± 47.55	219.22 ± 39.91	231.19 ± 40.48	233.59 ± 29.94
Phenylalanine (μmol/L)	132.78 ± 19.28	138.38 ± 8.49	139.81 ± 13.75	137.70 ± 12.99
Phe/Tyr (mol/mol)	0.61 ± 0.08	0.65 ± 0.10	0.62 ± 0.09	0.59 ± 0.06
TRP/AAA	0.36 ± 0.05	0.30 ± 0.04	0.33 ± 0.04	0.30 ± 0.03
TRP/LNAA	0.09 ± 0.01	0.09 ± 0.01	0.09 ± 0.01	0.09 ± 0.02
**HFP line**	**Undisrupted baseline PRE** **(*n* = 20)**	**Undisrupted POST** **(*n* = 20)**	**Disrupted baseline PRE** **(*n* = 20)**	**Disrupted POST** **(*n* = 20)**
Tryptophan (μmol/L)	105.58 ± 8.82	99.14 ± 12.74	108.34 ± 12.58	104.82 ± 9.65
Kynurenine (μmol/L)	0.35 ± 0.11	0.34 ± 0.08	0.37 ± 0.10	0.32 ± 0.07
Kyn/Trp (μmol/mmol)	32.7 ± 10.0	34.5 ± 6.8	34.2 ± 8.9	30.9 ± 6.0
Neopterin (nmol/L)	2.87 ± 0.54	3.21 ± 0.41	2.88 ± 0.48	3.17 ± 0.25
Tyrosine (μmol/L)	173.03 ± 17.57	165.95 ± 8.34	194.32 ± 32.74	189.17 ± 18.54
Phenylalanine (μmol/L)	130.98 ± 23.19	130.28 ± 25.82	135.10 ± 14.08	133.33 ± 9.42
Phe/Tyr (mol/mol)	0.73 ± 0.22	0.80 ± 0.11	0.71 ± 0.08	0.71 ± 0.09
TRP/AAA	0.39 ± 0.05	0.34 ± 0.04	0.37 ± 0.04	0.32 ± 0.03
TRP/LNAA	0.09 ± 0.01	0.08 ± 0.01	0.09 ± 0.01	0.08 ± 0.01

### Effects of Genetics and Social Disruption on FP Behavior and Body Weight

There was a significant effect of genetic line [*F*_(2, 152)_ = 53.30, *P* = 0.001], where HFP birds performed 68% more FP (8.6 ± 3.7) compared to LFP (2.7 ± 1.8; *t* = -7.38; *P* < 0.0001) and 44% more than UC birds (4.8 ± 1.8; *t* = -10.12; *P* < 0.0001). The occurrence of FP behavior (mean ± SD pecks per hour) was significantly affected by the social disruption treatment [*F*_(1, 152)_ = 20.58, *P* = 0.001], with socially disrupted groups showing 33 % more of FP than undisrupted groups (4.6 ± 3.8 vs. 3.1 ± 3.3) across all lines. There was a significant effect of social disruption treatment [*F*_(2, 153)_ = 6.36, *P* = 0.01] on body weight gain (384.36 ± 17.91 g) compared to non-disrupted groups (320.73 ± 17.75 g; *t* = -2.52, *P* < 0.0127) across all lines over a period of 3 weeks.

### Effects of Genetics and Social Disruption on Metabolism of Amino Acids

LFP birds (103.72 ± 2.17 μmol/L) had significantly higher blood TRP concentrations than UC birds (98.48 ± 1.25 μmol /L; *t* = -2.08; *P* < 0.04); however, LFP birds did not differ from HFP birds (101.77 ± 1.89 μmol/L). Social disruption treatment tended to increase blood TRP concentrations [*F*_(1, 152)_ = 3.4, *P* = 0.07], where TRP concentrations tended to be higher in socially disrupted (103.25 ± 1.48 μmol/L) than non-disrupted groups (99.4 ± 1.5 μmol/L; *t* = -84; *P* = 0.07) across all lines.

There was a significant effect of genetic line [*F*_(2, 152)_ = 8.03, *P* = 0.0005], which could be attributed to lower TRP/*Σ* AAA ratios in UC birds (0.288 ± 0.002) compared to LFP birds (0.3 ± 0.004; *t* = -1.97, *P* < 0.05] and HFP birds (0.311 ± 0.004 *t* = -3.92, *P* < 0.0001).

TRP/*Σ* LNAA concentrations were not affected by genetic line [HFP birds: 0.085 ± 0.001; LFP: 0.089 ± 0.0002; UC: 0.09 ± 0.001 5; *F*_(2, 152)_ = 1.3, *P* = 0.27], nor social disruption [disrupted: 0.088 ± 0.001 vs. non-disrupted: 0.086 ± 0.001; *F*_(1, 152)_ = 2.82, *P* = 0.09], nor interactions were observed for TRP/*Σ* LNAA concentrations.

There was a significant effect of line [*F*_(2, 152)_ = 17.27, *P* = 0.001], where HFP birds had 15 % higher PHE/TYR ratios (0.74 ± 0.01 μmol/ μmol) compared to LFP (0.63 ± 0.01 μmol/ μmol; *t* = -5.34; *P* < 0.0001) and 11% higher PHE/TYR ratios compared to UC birds (0.65 ± 0.001 μmol/ μmol ± 0.005; *t* = -5.13; *P* < 0.0001). There was a significant effect of social disruption treatment on PHE/TYR [*F*_(1, 152)_ = 15.51, *P* = 0.0001], where socially disrupted birds had 8.5% lower PHE/TYR ratios (0.65 ± 0.01 μmol/ μmol) compared to birds in non-social disrupted groups (0.7 ± 0.009 μmol/μmol; *t* = 3.94; *P* < 0.0001) across all lines.

### Effects of Genetics and Social Disruption on Biomarkers of Immune Activation

KYN concentrations were not affected by genetic line effects [HFP birds: 0.33 ± 0.03 μmol/L; LFP: 0.34 ± 0.04; UC: 0.37 ± 0.02 5; *F*_(2, 152)_ = 0.524, *P* = 0.59]; Table 2, norsocial disruption [disrupted: 0.35 ± 0.03 μmol/L vs. non-disrupted: 0.35 ± 0.03 μmol/L; *F*_(1, 152)_ = 0.00, *P* = 0.94], nor were interactions observed for KYN concentrations; Table 1.

HFP birds (3.2 ± 0.13 μmol/mmol), compared to UC birds (3.53 ± 0.08 μmol/ mmol; *t* = 2.15; *P* < 0.03), had significantly lower KYN/TRP ratios (9.35%); Table 2. There was a tendency of social disruption treatment on KYN/TRP [*F*_(1, 152)_ = 3.41, *P* = 0.07], where social disrupted birds had 7.43% lower KYN/TRP ratios (3.24 ± 0.1 μmol/ mmol) compared to birds in non-social disrupted groups (3.5 ± 0.099 μmol/ mmol; *t* = 1.85; *P* < 0.07) across all lines; Table 1.

Neopterin levels were not significantly different between lines; Table 2 (HFP birds: 2.88 ± 0.08 nmol/L, LFP birds: 2.78 ± 0.096 nmol/L, UC birds: 2.82 ± 0.05 nmol/L). Similarly, no significant differences were observed between neopterin levels in social disrupted (2.9 ± 0.06 nmol/L) and non-social disrupted (2.7 ± 0.06 nmol/L) groups; Table 1.

## Discussion

In the present study, we investigated the effect of social disruption stress in three chicken lines genetically selected for FP behavior and compared the FP response and TRP/KYN metabolic pathway. As far as we are aware, no previous study reported avian TRP degradation rate and FP behavior after a socially stressful situation in domestic birds, nor has the TRP/KYN pathway been compared between genetic lines selected for or against FP behavior.

The disruption of an established social environment produced an increase in FP behavior when compared to control groups. The social disruption treatments resulted in familiar birds being taken out and new birds being introduced into a one pen environment. These birds are both a resident group faced with newcomers and a new group coming into an existing group. FP in this context could reflect frustration (45, 46) after disruption of an established social environment (social bonds/relationships were built throughout the 16 weeks and then disrupted). Indeed, the exposure to social stressors is a critical risk factor for developing impaired social behavior in humans (47, 48) and other animals (49). However, from an evolutionary point of view, any negative/damaging social interactions after losing a social environment could also be interpreted as an adverse response to separation, which could encourage social animals to remain in a group (50). However, Riedstra and Groothuis (51) stated that FP could develop from a normal bird-to-bird social exploration, where chicks direct their pecks preferentially toward unfamiliar birds (52), which also helps to maintain a social relationship (53). It remains possible that the birds in our study developed pecking at the feather cover of other birds to socially explore unfamiliar birds, meaning a “normal basal level of allopreening” (54, 55). Both explanations, frustration, and social exploration, may have contributed to the increase in FP in the present study, but their relative importance and interaction are unknown.

Higher FP levels in HFP birds demonstrated the influence of genetic background on FP behavior. No interaction between genetic line and social disruption stress was found. This result was in contrast to our prediction. Social disruption stress did not make HFP birds more susceptible to developing FP behavior compared to LFP and UC birds, even though these genetic lines (18) differ on a behavioral and physiological level in stressful laboratory test situations in HPA-axis (56) and the relative sympathetic and parasympathetic nervous system activation responses (16).

Social disruption treatment tended to, and in LFP birds significantly, increase blood TRP levels. Blood levels of TRP are dependent on nutrition, metabolic rates and interactions between metabolites of amino acids, carbohydrates, and lipids (57). We cannot rule out that different amounts of feed consumed by certain individuals may have affected blood TRP levels. Interestingly, birds in disrupted groups gained more weight, likely due to increased feed intake, which might have impacted blood TRP levels in these birds. Stress can elevate brain TRP levels (23). The uptake of TRP into the brain is not determined by the absolute TRP concentrations, but rather by the ratio of TRP to other relevant amino acids. One mechanism regulating TRP uptake into the brain is by an elevation of the TRP/AAA or TRP/ LNAA ratios, which favor TRP uptake in the brain and thereby increases brain TRP levels (58) and potential TRP related behavior. However, we did not observe elevated TRP/AAA or TRP/LNAA ratios after social disruption treatment in the present study.

In addition to higher FP activity in social disrupted groups and HFP birds, birds in disrupted social environments tended to have lower KYN/TRP ratios, and HFP birds had significantly lower KYN/TRP ratios compared to UC birds, which was in contrast to our expectations. Lower KYN/TRP ratios may either indicate that blood TRP concentrations are kept more constant in HFP vs. UC birds, likely attributable to better food uptake, and that there is no immune activation which triggers downstream metabolism along the kynurenine axis. As neopterin, a marker of activation for the cellular immune system in human, was present at very low concentrations and did not differ among the bird lines, it remains questionable whether this marker can be used to indicate immune system activation in avians. If there is more TRP present in the system, this may point toward an increased formation of 5-HT along the serotonergic axis and away from KYN production.

The biological roles of KYN's downstream metabolites differ: KYN serves as an important precursor of the neuroprotective kynurenic acid, a glutamate receptor antagonist, however another downstream product, quinolinic acid is neurotoxic. Human patients with colorectal cancer, for example, often present with elevated KYN/TRP ratios, and the lowering of TRP due to its increased breakdown to KYN was found to be associated with depression (59).

However, the results obtained from this study cannot determine whether the KYN/TRP ratios impact FP or whether a higher number of FP pecks was caused by a lack of neuroprotective kynurenic acid.

The regulatory mechanisms used in TRP metabolism by domestic birds compared to humans may indicate major evolutionary differences in the breakdown of this molecule. For example, an NCBI GenBank Homologene search revealed *Gallus Gallus* orthologs for human IDO-2 and TDO, but not for IDO-1. IDO-2 has only 3–5% enzymatic activity of IDO-1 in humans (26). Therefore, a more detailed analysis of regulatory mechanisms is necessary to understand the role of TRP metabolism for laying hen immune system and brain function.

As we measured for the first time in our study in the domestic adolescent laying hen the KYN pathway of TRP metabolism, it is noteworthy that blood plasma concentrations of KYN measured in adolescent birds appear much lower (Tables 1, 2) than what has been found in human studies [1.78 ± 0.04, *n* = 100; see (60)] and TRP levels higher (Tables 1, 2) compared to humans [67.4 ± 1.02, *n* = 100; EM ± SE; values are taken from (60)].

For groups with social disruption treatment, PHE/TYR ratios were significantly lower than in the non-disrupted groups. PHE is an essential amino acid that can be converted to TYR, the precursor of catecholamines and thyroid hormones (35, 61). TYR has been shown to be an important amino acid for producing proteins, which participate in host defense activities, where their role of synthesis increases several folds under stress (62). Stressful events can increase PAH activity, decrease PHE/TYR ratios, and increase dopaminergic activity in mammals (63). Although we did not measure corticosterone as a marker for physiological stress response in the present study, higher corticosterone levels in FP birds shown in the study by van Hierden et al. (21), and the low PHE/TYR ratio in blood plasma of birds in social disrupted groups (and higher FP levels) could indicate a stress-mediated increase in PAH activity. Indeed, Birkl et al. (36) measured low PHE/TYR ratios in birds kept in groups with aggressive pecking/negative social interaction problems (psychological stressor) and in birds with an early onset of egg-laying (physiological stressor), suggesting a stress-mediated increase in PAH. This observation is also confirmed by Dennis and Cheng (64), suggesting a link between high egg production and aggressive behavior in laying hens selected for survivability and production (65), where the dopaminergic system is likely impacted by reproductive physiology. Interestingly, lower PHE/TYR ratios in athletes performing exhaustive exercises were interpreted as more probable to produce higher catecholamine levels, which may compensate, at least partly, for mood lowering intensive exercise (stressor) programs (66). Whether birds in socially disrupted groups with lower PHE/TYR ratios produce more catecholamines and increase brain dopaminergic activity needs further investigation.

The development of body weight was significantly affected by social disruption treatment; surprisingly, birds in socially disrupted groups gained more weight. In humans, facing stressors might increase or decrease food intake (67). In the case of chronic stress or if there are multiple sequential stressors, glucocorticoid levels can be maintained chronically high, leading to increased feeding and consequent obesity (68). We did not measure feed intake in the present study. Nevertheless, the weight gain in socially disrupted birds could indicate higher feed consumption. Considering the social disruption treatment has strong adverse effects on psychological (increased FP) and physiological (amino acid metabolism) functioning, birds could have consumed more food, as shown in rat models for obesity research (69). In mammals, palatable food can activate the dopaminergic pathway where high levels of dopamine help to extinguish the activity of the HPA axis (70). Similar considerations could be hypothesized in the present study: Social disruption treatment triggered pecking at the feather cover of other birds, where birds might pluck and eat feathers (3, 71) leading to positive post-ingestive feedback and making feathers highly palatable (40). Additionally, feather ingestion increases metabolism efficiency by increasing conversion of feed into body weight (72). Together, the highly palatable feathers and glucocorticoids (high pen specific stress levels in socially disrupted groups) might have activated the dopaminergic circuit reward system (they cannot or will not stop), indicated by the low PHE/TYR ratio, to peck at, pluck and eat palatable feathers and feed. Despite this hypothesis, a remaining question is why genetically selected HFP birds (susceptible to FP) showed higher PHE/TYR ratios when we would expect them to be more prone toward engaging in FP and in this positive feedback-loop.

Avian plasma neopterin concentrations could be detected in adolescent birds, albeit at much lower concentrations than in humans. Even so, neither social disruption treatments nor genetic differences between lines impacted neopterin levels. Though neopterin as an immune marker in avian species would have been fortunate, as avian immune activation is often difficult to assess due to the limited availability of cytokine detection assays, our results point toward a different regulation of the pteridine metabolism in the avian and human systems.

A potential limitation of the present study is that the measured peripheral plasma blood levels of amino acids and their specific metabolites might not be representative of their levels in the CNS. However, it has been shown in human and other mammals that there are strong associations between blood plasma and cerebrospinal fluid levels of KYN/TRP and PHE/TYR (73, 74). Strikingly, Kops et al. (75) showed that HFP birds have higher 5-HT and dopaminergic turnover in the limbic caudolateral nidopallium of the avian brain, which serves comparable functions to the mammalian prefrontal cortex (76). However, we are not aware of any investigation investigating brain TRP/KYN metabolism in relation to FP. A further limitation of our study is that we cannot rule out that feed/water consumption may have affected the metabolite levels.

Additionally, it should be stated that the severity of FP observed in these adolescent birds was moderate, in that we did not observe significant damage to the feather-cover. Also, Piepho et al. (77) described “extreme” feather peckers as individuals who performed severe FP consistently in at least 3 out of 5 observations. This could not be observed in our study. The moderate FP level which we observed might be explained in part by the fact that these birds were relatively young (16 weeks of age) and that birds were kept in enriched “luxury” environments (elevated platforms, perches, nest boxes), compared to commercial housing systems, where birds could avoid negative social interactions. Also, the stress level induced in the experiment is likely much less than that in effect in commercial situations (large groups and high stocking densities), in terms of severity and duration, which might have impacted behavioral and physiological measurements. Worldwide, the majority of birds are reared on pullet (birds until sexual maturity; before egg laying) farms being transferred to egg layer farms for production. This transition between physical and social environments can be drastic and could lead to physiological changes which result in FP. However, birds in the present study were given the opportunity to have a fixed social environment for the first 16 weeks of life, in a relatively small group. In comparison, commercial situations will not permit any fixed social environment throughout their entire life, due to large group size and disruption between rearing and laying. So, social stress may have a more chronic and severe impact on commercial birds compared to the birds of this study. Furthermore, the number of pens per treatment was relatively small, and larger studies are needed to confirm the results.

In conclusion, as hypothesized, birds in social disrupted groups showed a higher FP activity. KYN and neopterin concentrations could be detected in laying hen blood. TRP, PHE, TYR, and their relevant ratios could be used to differentiate birds in social disrupted from undisrupted groups. However, contrary to our expectation, birds in social disrupted environments tended to have lower PHE/TYR ratios, higher TRP concentrations and lower KYN/TRP ratios; lower KYN/TRP ratios were especially unexpected. Therefore, monitoring the avian amino acid metabolism and how it impacts the etiology and physiology of FP in laying hens might offer new avenues to understand this behavioral problem.

## Data Availability

The datasets generated for this study are available on request to the corresponding author.

## Ethics Statement

This study was approved by the University of Guelph Animal Care Committee before testing (Animal Utilization Protocol # 3206).

## Author Contributions

AH-M, PB, JK, WK, and PF conceived and designed the study. PB, JC, and PM carried out the study. JC and PM analyzed the videos. JG and DF analyzed the blood samples. PB analyzed the data and drafted the manuscript. AH-M, JC, PM, JK, WK, PF, JG, and DF contributed to writing the manuscript. All authors read and approved the final manuscript.

### Conflict of Interest Statement

The authors declare that the research was conducted in the absence of any commercial or financial relationships that could be construed as a potential conflict of interest.

## Abbreviations

FP: Feather Pecking
HFP: High Feather Pecking Behavior
LFP: Low Feather Pecking Behavior
UC: Unselected Control Line.

## References

[1] Blokhuis HJ Van Niekerk TF Bessei W Elson A Guémené D Kjaer JB The LayWel project: welfare implications of changes in production systems for laying hens. World Poultry Sci J. (2007) 63:101–14. 10.1017/S0043933907001328

[2] Neal WN Cannibalism, pick-outs and methionine. Poultry Sci. (1956) 35:10–3. 10.3382/ps.0350010

[3] Harlander-Matauschek A Häusler K Bessei W A note on the relative preferences of laying hens for feathers from different body parts. Appl Anim Behav Sci. (2007) 108:186–90. 10.1016/j.applanim.2006.11.010

[4] Harlander-Matauschek A Wassermann F Zentek J Bessei W. Laying hens learn to avoid feathers. Poultry Sci. (2008) 87:1720–4. 10.3382/ps.2007-00510 18753438

[5] Harlander-Matauschek A Feise U Physical characteristics of feathers play a role in feather-eating behaviour. Poultry Sci. (2009) 88:1800–4. 10.3382/ps.2008-00566 19687262

[6] Savory CJ Feather pecking and cannibalism. World Poultry Sci J. (1995) 51:215–9. 10.1079/WPS19950016

[7] van Staaveren N Decina C Baes CF Widowski TM Berke O Harlander-Matauschek A. A description of laying hen husbandry and management practices in Canada. Animals. (2018) 8:114. 10.3390/ani8070114 29997334 PMC6071255

[8] McBride G Parer IP Foenander F The social organization and behaviour of the feral domestic fowl. Anim Behav Monogr. (1969) 2:125–81. 10.1016/S0066-1856(69)80003-8

[9] Wood-Gush DGM. The Behavior of the Domestic Fowl. London: Heinemann Educational (1971).

[10] Bilčik B Keeling LJ. Relationship between feather pecking and ground pecking in laying hens and the effect of group size. Appl Anim Behav Sci. (2000) 68:55–66. 10.1016/S0168-1591(00)00089-7 10771315

[11] Cheng HW Muir WM. Chronic social stress differentially regulates neuroendocrine responses in laying hens: II. Genetic basis of adrenal responses under three different social conditions. Psychoneuroendocrinology. (2004) 29:961–97. 10.1016/j.psyneuen.2003.09.002 15177713

[12] Cheng HW Singleton P Muir WM. Social stress in laying hens: differential dopamine and corticosterone responses after intermingling different genetic strains of chickens. Poultry Sci. (2002) 81:1265–72. 10.1093/ps/81.9.1265 12269602

[13] Birkl P Chow J McBride P Forsythe P Kjaer JB Kunze W Effects of acute tryptophan depletion on repetitive behavior in laying hens. Front Vet Sci. (2018). 10.3389/fvets.2019.00230 PMC6637846 31355217

[14] Hughes BO Duncan IJH. The influence of strain and environmental factors upon feather pecking and cannibalism in fowls. Brit Poultry Sci. (1972) 13:525–47. 10.1080/00071667208415981 4638817

[15] Jones RB Hocking PM Genetic selection for poultry behaviour: big bad wolf or friend in need? Anim Welfare. (1999) 8:343–59.

[16] Kjaer JB Jørgensen H. Heart rate variability in domestic chicken lines genetically selected on feather pecking behavior. Genes Brain Behav. (2011) 10:747–55. 10.1111/j.1601-183X.2011.00713.x 21682845

[17] De Haas N van der Eijk JA. Where in the serotonergic system does it go wrong? Unravelling the route by which the serotonergic system affects feather pecking in chickens. Neurosci Biobehav R. (2018) 95:170–88. 10.1016/j.neubiorev.2018.07.007 30055196

[18] Kjaer JB Sørensen P Su G. Divergent selection on feather pecking behaviour in laying hens (Gallus gallus domesticus). Appl Anim Behav Sci. (2001) 71:229–39. 10.1016/S0168-1591(00)00184-2 11230903

[19] Biscarini F Bovenhuis H Van der Poel J Rodenburg TB Jungerius AP Van Arendonk JAM. Across-line SNP association study for direct and associative effects on feather damage in laying hens. Behav Genet. (2010) 40:715–27. 10.1007/s10519-010-9370-0 20496162

[20] van Hierden YM Korte SM Ruesink EW van Reenen CG Engel B Korte-Bouws GA. Adrenocortical reactivity and central serotonin and dopamine turnover in young chicks from a high and low feather-pecking line of laying hens. Physiol Behav. (2002) 75:653–9. 10.1016/S0031-9384(02)00667-4 12020730

[21] van Hierden YM Koolhaas JM Korte SM Chronic increase of dietary L-tryptophan decreases gentle feather pecking behaviour. Appl Anim Behav Sci. (2004) 89:71–84. 10.1016/j.applanim.2004.05.004

[22] Savory CJ Mann JS MacLeod MG Incidence of pecking damage in growing bantams in relation to food from, groups size, stocking density, dietary tryptophan concentration and dietary protein source. Brit Poultry Sci. (1999) 40:579–84. 10.1080/00071669986936 10670667

[23] Miura H Ozaki N Sawada M Isobe K Ohta T Nagatsu T. A link between stress and depression: shifts in the balance between kynurenine and serotonin pathways of tryptophan metabolism and the etiology and pathophysiology of depression. Stress. (2008) 11:198–209. 10.1080/10253890701754068 18465467

[24] Widner B Laich A Sperner-Unterweger B Ledochowski M Fuchs D. Neopterin production, tryptophan degradation, and mental depression-What is the link?. Brain Behav Immun. (2002) 16:590–5. 10.1016/S0889-1591(02)00006-5 12401473

[25] Bryn V Verkerk R Skjeldal O Saugstad OD Ormstad H. Kynurenine pathway in autism spectrum disorders in children. Neuropsychobiology. (2018) 76:1–7. 10.1159/000488157 29694960

[26] Ball HJ Sanchez-Perez A Weiser S Austin CJ Astelbauer F Miu J. Characterization of an indoleamine 2,3-dioxygenase-like protein found in humans and mice. Gene. (2007) 396:203–13. 10.1016/j.gene.2007.04.010 17499941

[27] Oxenkrug GF. Tryptophan-kynurenine metabolism as a common mediator of genetic and environmental impacts in major depressive disorder: the serotonin hypothesis revisited 40 years later. Isr J Psychiatry Relat Sci. (2010) 47:56–63. 20686200 PMC3021918

[28] Stone TW Stoy N Darlington LG. An expanding range of targets for kynurenine metabolites of tryptophan. Trends Pharmacol Sci. (2013) 34:136–43. 10.1016/j.tips.2012.09.006 23123095

[29] Konsman JP Parnet P Dantzer R. Cytokine-induced sickness behaviour: mechanisms and implications. Trends Neurosci. (2002) 25:154–9. 10.1016/S0166-2236(00)02088-9 11852148

[30] Leathwood PD Fernstrom JD. Effect of an oral tryptophan/carbohydrate load on tryptophan, large neutral amino acid, and serotonin and 5-hydroxyindoleacetic acid levels in monkey brain. J Neural Transm Gen Sect. (1990) 79:25–34. 10.1007/BF01250997 1688706

[31] Markus CR. Dietary amino acids and brain serotonin function; implications for stress-related affective changes. NeuroMol Med. (2008) 10:247–58. 10.1007/s12017-008-8039-9 18516508

[32] Fuchs D Möller AA Reibnegger G Werner ER Werner-Felmayer G Dierich MP. Increased endogenous interferon-gamma and neopterin correlate with increased degradation of tryptophan in human immunodeficiency virus type 1 infection. Immunol Lett. (1991) 28:207–11. 10.1016/0165-2478(91)90005-U 1909303

[33] Kurz K Schroecksnadel S Weiss G Fuchs D. Association between increased tryptophan degradation and depression in cancer patients. Curr Opin Clin Nutr Metab Care. (2011) 14:49–56. 10.1097/MCO.0b013e328340d849 21076293

[34] Umehara H Numata S Watanabe SY Hatakeyama Y Kinoshita M Tomioka Y. Altered KYN/TRP, Gln/Glu, and Met/methionine sulfoxide ratios in the blood plasma of medication-free patients with major depressive disorder. Sci Rep. (2017) 7:4855. 10.1038/s41598-017-05121-6 28687801 PMC5501805

[35] Neurauter G Schrocksnadel K Scholl-Burgi S Sperner-Unterweger B Schubert C Ledochowski M. Chronic immune stimulation correlates with reduced phenylalanine turnover. Curr Drug Metab. (2008) 9:622–7. 10.2174/138920008785821738 18781914

[36] Birkl P Franke L Rodenburg TB Ellen E Harlander-Matauschek A. A role for plasma aromatic amino acids in injurious pecking behavior in laying hens. Physiol Behav. (2017) 175:88–96. 10.1016/j.physbeh.2017.03.041 28365278

[37] Fuchs D Weiss G Wachter H. Neopterin, biochemistry and clinical use as marker for cellular immune reactions. Int Arch Allergy Immunol. (1993) 101:1–6. 10.1159/000236491 8499767

[38] Segerstrom SC Miller GE. Psychological stress and the human immune system: a meta-analytic study of 30 years of inquiry. Psychol Bull. (2004) 130:601–30. 10.1037/0033-2909.130.4.601 15250815 PMC1361287

[39] Kozak M Tobalske B Martins C Bowley S Wuerbel H Harlander-Matauschek A Use of space by domestic chicks housed in complex aviaries. Appl Anim Behav Sci. (2016) 181:115–21. 10.1016/j.applanim.2016.05.024

[40] Harlander-Matauschek A Beck P Piepho HP Taste aversion learning to eliminate feather pecking in laying hens, *Gallus gallus domesticus*. Anim Behav. (2009) 78:485–90. 10.1016/j.anbehav.2009.05.020

[41] Kjaer JB Vestergaard KS Development of feather pecking in relation to light intensity. Appl Anim Behav Sci. (1999) 62:243–54. 10.1016/S0168-1591(98)00217-2

[42] Altmann J. Observational study of behavior: sampling methods. Behavior. (1974) 49:227–66. 10.1163/156853974X00534 4597405

[43] Neurauter G Scholl-Bürgi S Haara A Geisler S Mayersbach P Schennach H. Simultaneous measurement of phenylalanine and tyrosine by high performance liquid chromatography (HPLC) with fluorescence detection. Clin Biochem. (2013) 46:1848–51. 10.1016/j.clinbiochem.2013.10.015 24183885

[44] Birkl P McBride P Forsythe P Kjaer JB Kunze W Fuchs D Comparing plasma tryptophan measurements between labs using UPLC/HPLC. Pteridines. (2018) 29:42–3. 10.1515/pteridines-2018-0006

[45] Grigor PN Hughes BO Appleby MC Social inhibition of movement in domestic hens. Anim Behav. (1995) 49:1381–8. 10.1006/anbe.1995.0168

[46] Lindberg AC Nicol CJ Effects of social and environmental familiarity on group preferences and spacing behaviour in laying hens. Appl Anim Behav Sci. (1996) 49:109–23. 10.1016/0168-1591(96)01046-5

[47] Van der Kolk BA. The neurobiology of childhood trauma and abuse. Child Adolesc Psychiatr Clin N Am. (2003) 12:293–317. 10.1016/S1056-4993(03)00003-8 12725013

[48] Widom CS Maxfield MG. A prospective examination of risk for violence among abused and neglected children. Ann NY Acad Sci. (1996) 794:224–37. 10.1111/j.1749-6632.1996.tb32523.x 8853605

[49] El-Lethey H Aerni V Jungi TW Wechsler B. Stress and feather pecking in laying hens in relation to housing conditions. Brit Poultry Sci. (2000) 41:22–8. 10.1080/00071660086358 10821518

[50] Alexander R The evolution of social behavior. Ann Rev Ecol Syst. (1974) 5:325–83. 10.1146/annurev.es.05.110174.001545

[51] Riedstra B Groothuis TGG Early feather pecking as a form of social exploration: the effect of group stability on feather pecking and tonic immobility in domestic chicks. Appl Anim Behav Sci. (2002) 77:127–38. 10.1016/S0168-1591(02)00031-X

[52] Zajonc RB Wilson WR Rajecki DW. Affiliation and social discrimination produced by brief exposure in day-old domestic chicks. Anim Behav. (1975) 23:131–8. 10.1016/0003-3472(75)90059-7 1155813

[53] Wood-Gush DGM Rowland CG Allopreening in the domestic fowl. Rev Comp Animal. (1973) 7:83–91.

[54] Blokhuis HJ Feather - pecking in poultry: its relation with ground-pecking. Appl Anim Behav Sci. (1986) 16:63–7. 10.1016/0168-1591(86)90040-7

[55] Vestergaard KS Kruijt JP Hogan JA Feather pecking and chronic fear in groups of red jungle fowl: their relations to dustbathing, rearing environment and social status. Anim Behav. (1993) 45:1127–40. 10.1006/anbe.1993.1137

[56] Kjaer JB Guémené D. Adrenal reactivity in lines of domestic fowl selected on feather pecking behavior. Physiol Behav. (2009) 96:370–3. 10.1016/j.physbeh.2008.10.023 19027766

[57] Gannon MC Nuttall FQ. Amino acid ingestion and glucose metabolism: a review. Life. (2010) 62:660–8. 10.1002/iub.375 20882645

[58] Fernstrom JD Wurtman R. Brain serotonin content: physiological regulation by plasma neutral amino acids. Science. (1972) 178:414–16. 10.1126/science.178.4059.414 5077329

[59] Huang A Fuchs D Widner B Glover C Henderson DC Allen-Mersh TG. Serum tryptophan decrease correlates with immune activation and impaired quality of life in colorectal cancer. Br J Cancer. (2002) 86:1691–6. 10.1038/sj.bjc.6600336 12087451 PMC2375406

[60] Geisler S Mayersbach P Becker K Schennach H Fuchs D Gostner JM Serum tryptophan, kynurenine, phenylalanine, tyrosine and neopterin concentrations in 100 healthy blood donors. Pteridines. (2015) 26:31–6. 10.1515/pterid-2014-0015

[61] Haroon E Raison CL Miller AH. Psychoneuroimmunology meets neuropsychopharmacology: translational implications of the impact of inflammation on behavior. Neuropsychopharmacology. (2012) 37:137–62. 10.1038/npp.2011.205 21918508 PMC3238082

[62] Jahoor F Baddaloo A Reid M Forrester T. Glycine production in severe childhood undernutrition. Am J Clin Nutr. (2006) 84:143–9. 10.1093/ajcn/84.1.143 16825688

[63] Pani I Porcella A Gessa GL. The role of stress in the pathophysiology of the dopaminergic system. Mol Psychiatry. (2000) 5:14–21. 10.1038/sj.mp.4000589 10673764

[64] Dennis R Cheng HW. The dopaminergic system and aggression in laying hens. Poultry Sci. (2011) 90:2440–8. 10.3382/ps.2011-01513 22010227

[65] Cheng H Muir W Mechanisms of aggression and production in chickens: genetic variations in the functions of serotonin, catecholamine, and corticosterone. World's Poultry Sci J. (2007) 63:233–54. 10.1017/S0043933907001432

[66] Strasser B Geiger D Schauer M Gatterer H Burtscher M Fuchs D. Effects of exhaustive aerobic exercise on tryptophan-kynurenine metabolism in trained athletes. PLoS ONE. (2016) 11:e0153617. 10.1371/journal.pone.0153617 27124720 PMC4849644

[67] Demori I Grasselli E Stress-related weight gain: mechanisms involving feeding behavior, metabolism, gut microbiota and inflammation. J Nutr Food Sci. (2016) 6:1–6. 10.4172/2155-9600.1000457

[68] Spencer SJ Tillbrook A. The glucocorticoid contribution to obesity. Stress. (2011) 14:233–46. 10.3109/10253890.2010.534831 21294656

[69] Pecoraro N Reyes F Gomez F Bhargava A Dallman MF. Chronic stress promotes palatable feeding, which reduces signs of stress: feedforward and feedback effects of chronic stress. Endocrinology. (2004) 145:3754–62. 10.1210/en.2004-0305 15142987

[70] Volkow ND Wang GJ Baler RD. Reward, dopamine and the control of food intake: implications for obesity. Trends Cogn Sci. (2011) 15:37–46. 10.1016/j.tics.2010.11.001 21109477 PMC3124340

[71] Harlander-Matauschek A Baes C Bessei W The demand of laying hens for feathers and wood shavings. Appl Anim Behav Sci. (2006) 101:102–10. 10.1016/j.applanim.2006.01.003

[72] Harlander-Matauschek A Bessei W Feather eating and crop filling in laying hens. Arch Geflugelkd. (2005) 69:241–4.

[73] Curzon G Relationships between plasma, CSF, and brain tryptophan. In: Baumann P editor. Transport Mechanisms of Tryptophan in Blood Cells, Nerve Cells, and at the Blood-Brain Barrier, Journal of Neural Transmission, Volume 15 Vienna: Springer (1979). 10.1007/978-3-7091-2243-3_7 290764

[74] Gal EM Sherman AD. L-kynurenine: its synthesis and possible regulatory function in brain. Neurochem Res. (1980) 5:223–39. 10.1007/BF00964611 6154900

[75] Kops MS Kjaer JB Güntürkün O Westphal KG Korte-Bouws GA Olivier B. Brain monoamine levels and behaviour of young and adult chickens genetically selected on feather pecking. Behav Brain Res. (2017) 327:11–20. 10.1016/j.bbr.2017.03.024 28347825

[76] Herold C Palomero-Gallagher N Hellmann B Kröner S Theiss C Güntürkün O. The receptorarchitecture of the pigeons' nidopallium caudolaterale: an avian analogue to the mammalian prefrontal cortex. Brain Struct Funct. (2011) 216:239–54. 10.1007/s00429-011-0301-5 21293877

[77] Piepho HP Lutz V Kjaer JB Grashorn M Bennewitz J Bessei W. The presence of extreme feather peckers in groups of laying hens. Animal. (2017) 11:500–6. 10.1017/S1751731116001579 27476320

